# Stigmatisation of survivors of political persecution in the GDR: attitudes of healthcare professionals

**DOI:** 10.3389/fpsyt.2025.1556411

**Published:** 2025-06-10

**Authors:** Tobias Schott, Marie Blume, Anne Weiß, Christian Sander, Georg Schomerus

**Affiliations:** Department of Psychiatry and Psychotherapy, University of Leipzig Medical Centre, Leipzig, Germany

**Keywords:** GDR, SED, reunification, mental disorders, attitude, stigma, marginalization, health-care system

## Abstract

**Introduction:**

People with mental disorders face various barriers on the road to treatment. People who have experienced injustice of the state apparatus of the German Democratic Republic (GDR) in the form of various reprisals are a group that has received insufficient attention in research. Some of them still show long-term psychological and physical consequences that occur more frequently than in the general population, resulting in an increased need for treatment. There are currently no studies on how those affected are perceived by practitioners due to their history and whether they are exposed to stigmatizing attitudes.

**Method:**

A vignette-based survey was carried out to identify possible stigmatising attitudes. An independent opinion and survey institute conducted the study in three phases in December 2022, April 2023 and May to August 2024 using an online survey. A total of *N*=1357 practitioners from the German healthcare system were presented one of four case vignettes. The two vignettes described a person with mental health difficulties who had either experienced an unremarkable socialization in the GDR (A) or had suffered injustice in the GDR (B). In addition to socio-demographic variables, stereotypes, emotional reactions and desire for social distance towards the person described were recorded.

**Results:**

Age and sex as well as subjective knowledge about the GDR, the occupational group and the working environment influence the intensity of emotional reactions as well as the desire for social distance and the extent of negative stereotypical attitudes. The presentation of a case vignette that deals with an experience of SED injustice favours a decrease in positive and an increase in negative stereotypes. The explanatory power of the regression models is predominantly in the medium range (from 3.7 till 32.3%).

**Conclusions:**

Even more than three decades after the reunification of Germany, people with mental health problems and an experience of SED injustice in the GDR still experience stigmatizing attitudes on the part of those treating them. Stigmatizing attitudes can affect treatment and care.

## Introduction

Over the last three decades the prevalence rates of mental illness have risen continually (1) which is one of the reasons for significant costs for the society and health-system (2). Despite the increase in prevalence rates for mental disorders, there is a large discrepancy between the need and the actual care for various mental disorders, e.g., mood disorders, anxiety disorders or schizophrenia worldwide (3, 4). A small amount of individuals with a clinically relevant mental disorder actually receive appropriate treatment (5–7).

### The stigma of mental illness

In recent decades, while the scientific focus on the stigma of mental illness has increased, the stigma of mental illness still remains a major issue as a form of social injustice (8). The stigma model by Link and Phelan (9) comprises four interrelated components: Labelling, Stereotyping, Segregation, and Discrimination (9). A later extension to the model added an additional component, the emotional response, which stands between exclusion and discrimination (10). An imbalance of power is important in the process of discrimination, as only then stigmatization takes place (9). Stigma can negatively affect people in a number of ways: a person can stigmatize themselves and be stigmatized by the public (11) or be excluded by structural barriers (see section below). All three types of stigmatization have extensive consequences.

### Consequences of stigma regarding mental health

Several reviews show that both internalized and self-stigma have negative effects in people with mental illness, including lower self-efficacy, lower self-confidence, more severe symptoms, lower treatment adherence and lower quality of life (12–14). These factors, in turn, are unfavourable predictors for treatment admission and the further direction of treatment (for an overview see (15, 16)). In countries with strong structural stigmatization, people from minority groups have poorer mental health and greater physiological dysregulation (17, 18). Relatives of people with schizophrenia also suffer from inadequate structural support, as they need more support and are often under involved in the treatment process or have few support services (in sense of structural barriers (19–21)). In addition, population surveys show that financial support for research and care of mental illness is of low priority, compared to somatic illness (22, 23). Public stigma has also been shown to have long-term effects on self-stigma (24), and some studies suggest that public stigma indirectly influences helping behaviour via self-stigma (25–27).

### Experience of injustice in the German Democratic Republic and the consequences for those affected

The division of Germany (1949-1989/90) into the Federal Republic of Germany (*FRG*) and the German Democratic Republic (*GDR*) is an important part of German history. The GDR was a one-party dictatorship (*SED; Sozialistische Einheitspartei Deutschland*) that used various methods of political repression against deviants and political dissidents (28). Maercker and Guski-Leinwand (28) provide a comprehensive overview of the structure and methods of repression and persecution by the Ministry for State Security *(e.g., operative psychology as a means of applied psychology for secret police purposes, including interrogation, subversion and recruitment of ‘informal collaborators’)*. Furthermore, there were repeated individual cases in which psychiatry was misused for political purposes, for example to imprison socially undesirable people (29, 30). One of the methods used during this time was the political imprisonment of dissidents or people who tried to escape from the GDR.

Some Studies show the negative impact of unlawful incarcerations on mental health outside of the GDR context. In a review article by Brooks and Greenberg (31), twenty articles on the psychological consequences of false imprisonment were compiled. Eight themes emerged: loss of identity or personality changes, stigma and exclusion and self-stigmatization, mental and physical health problems such as depression, anxiety disorders, PTSD and sleep disorders, relationship problems (isolation, turning away from important family members), negative attitudes towards the justice system, financial consequences (e.g. through loss of income), traumatic experiences and adjustment difficulties after release. Studies on politically persecuted groups (e.g., tortured Turkish political activists, arrested and tortured Tibetan refugees) show that people who have experienced torture have significantly higher levels of symptoms of depression, anxiety and post-traumatic stress disorder compared to a control group (32–36).

These findings can also be transferred to people who suffered various forms of political repression under the SED regime. Research shows that people who experienced injustice in the GDR have an increased risk of somatic and mental disorders (37–40).

A study by Compera and colleagues (41) of people who experienced residential children’s home in the former GDR shows that more than two thirds of the respondents who were ever treated for a mental illness used psychotherapy, psychiatrist or inpatient treatment. In comparison, around 25% of patients with one clinically relevant diagnosis ever seek treatment. For patients with two diagnoses, this proportion is just under a third, while around 50% of patients with three diagnoses seek treatment (42). This shows that those affected visit several treatment services during the duration of their illness possibly due to a more intensive need for treatment. In addition, studies in German-speaking countries show that those affected by residential children’s home in the former GDR (43) have difficulties receiving support due to structural barriers to psychotherapeutic and somatic care (44). The number of unreported cases of people affected by injustice in the GDR could indicate that the need of adequate treatment is even higher. There seem to be further barriers in addition to the known obstacles.

It has also been shown that healthcare professionals who work with people who experienced residential care in the former GDR are confronted with certain challenges in treatment. These include, for example, difficulties in building relationships, complications in the process of treatment and insufficient confrontation with difficult events. There are also barriers such as fear of re-traumatisation, scepticism towards authorities, limited access to treatment services and a lack of knowledge on the part of those providing treatment (45). In addition, those affected by residential children’s home in the GDR reported various barriers and challenges in the context of treatment, such as feelings of shame, lack of trust in the treatment setting or concerns that the treatment providers did not have enough background knowledge (41, 46). These studies clearly state that those affected by experiences of injustice in the GDR have an increased need for treatment and that the practitioners need to be sensitive in the treatment.

### Potential stigmatization of individuals with SED injustice experience in the health care system

Previous studies have shown that people who experienced injustice in the GDR suffer from an increased psychophysiological stress. The experiences in the dictatorship and the various methods used by the Ministry for State Security of the GDR can generally lead to increased mistrust in the healthcare system. In a similar context, some studies show that racism or discrimination due to HIV disease as a form of structural discrimination leads to greater mistrust of the healthcare system (47, 48). This can lead to problems with health care seeking, as those people may find it difficult to open up in a potential treatment and may be perceived as demanding. However, there are no studies on how people with experiences of SED injustice are treated in the healthcare system and whether there are stigmatizing attitudes from healthcare providers towards this treatment group. According to a study by Hoffmann and colleagues (45) only around a quarter of the interviewed healthcare workers had a good or very good knowledge about the GDR. The lack of background knowledge can influence the diagnostic- and treatment process (45) and less knowledge is also associated with higher negative attitudes (49, 50). Stereotypes, as a part of the stigma process play an important role as a link between emotional reactions and subsequent discriminatory behaviour (10, 25). Comparable assumptions can also be found in the concept of public stigmatization by Corrigan and Watson (51). In this model prejudice which is accompanied by an emotional reaction, precedes the activation of stereotypes, which in turn are the precursor to discrimination. Another model is the empirically well-studied attribution theory by Weiner (52). Weiner’s attribution model (53) describes how assumptions about a person’s controllability and responsibility (stereotypes) evoke emotional reactions such as pity or anger, which in turn influence potential helping behaviour. A meta-analysis by Rudolph and colleagues (54) confirms this connection empirically.

Some studies indicate that more anger and less compassion is shown towards people suffering from diseases with a high degree of personal responsibility (e.g. AIDS, drug addiction as a result of risky behavior) than other diseases such as tuberculosis or Alzheimer’s disease (55). Various studies show that people have more negative attitudes towards groups of people if they attribute increased responsibility for certain characteristics or conditions to them. These include, for example, one’s own sexual orientation [homosexual vs. heterosexual (56)], obesity (57) or the development of a somatic or mental illness (58, 59).

### Research questions

The aim of this study is to investigate whether stereotypes influence attitudes of helping others in professionals of the health care system. Two vignettes were used to assess these stereotypes. The stability (high stability) was the same for both vignettes. We varied the causal and controllability attribution: internal and controllable (experience of injustice with high personal responsibility in the form of an escape) versus external and uncontrollable (low personal responsibility in a fatal car accident). The theoretical assumption is that an external and uncontrollable attribution leads to a lower emotional response accompanied by increased help-seeking behaviour by the professional or to less rejection (less stigmatizing attitudes) on the part of those treating the patient.

1. Do people who experienced SED injustice in the GDR face more stigmatizing attitudes from practitioners than people without such experiences?
2. Are there socio-demographic variables (age, sex, occupation, treatment setting, knowledge about the GDR, contact with whom, own experience of injustice) that are associated with stigmatizing attitudes towards people with experiences of SED injustice?

## Materials and methods

The quantitative study was conducted in the outpatient and inpatient healthcare sector. The aim was to assess the attitudes of healthcare professionals towards certain groups of people (people with experience of injustice under the SED). A case vignette-based approach was used for this purpose. Standardised case vignettes were used to measure possible stigmatising reactions to the people described.

### Sample

In order to achieve a wide reach and to include groups of people outside of academic or institutional contexts, the data was collected anonymously and voluntarily via a browser-based platform provided by the indepent opinion and survey institute Bilendi & Respondi (Berlin). Registered participants from the panel were invited to take part in the survey within the platform. To be eligible to participate, participants were asked in preliminary questions whether they were currently or had been working in the outpatient or inpatient healthcare sector for at least one year. They were also asked whether they belong to one of the following healthcare professions: medical doctors, nurses, speech therapists, physiotherapists, occupational therapists, psychologists or psychotherapists. They were also asked whether they had had direct patient contact in the past or were currently in contact with patients.

The independent opinion and survey institute Bilendi & Respondi (Berlin/Cologne) carried out the survey, which was conducted in three phases (December 2022, April 2023, Mai to August 2024). The participants were informed in advance about the objectives and methodology of the survey and gave their consent to participate before the survey began. In total, the survey institute provided a data set with *N* =1357 participants (male=652, female=705). The average age of participants was 43.14 years (*SD*=10.86). The sample is largely balanced in terms of sex, age, and federal states due to predefined quotas. Further socio-demographic variables are shown in **Table 1**.

**Table 1:** Sociodemographic variables.

Variable	Mean *(SD)* or % (*n*) (*N*=1357)
Age (years)	43.14 (10.86)
Sex	
Male	48.0% (652)
Female	52.0% (705)
Primarly socialization	
West-Germany	60.9% (826)
East-Germany	34.7% (471)
Outside of Germany	4.4% (60)
Professional group	
Medical doctor	8.7% (118)
Healthcare and nursing assistant	64.4% (874)
Psychologist/Psychotherapist	14.7% (200)
Other (Occupational therapist, Speech therapist, Physiotherapist, etc.)	12.2% (165)
Work setting	
Outpatient	45.2% (614)
Inpatient	54.8% (743)
Contact with people with experience of SED injustice	
Yes	28.6% (388)
No	71.4% (969)
Own experience of SED injustice in the GDR	
Yes	3.1% (42)
No	96.9% (1315)
ERMIS	
Fear	1.93 (0.99)
Anger	1.81 (1.01)
Pro-social behavior	3.63 (0.78)
Social Distance Scale	2.63 (0.77)
Positive Stereotype	3.53 (0.68)
Negative Stereotype	2.83 (0.69)
Subjective Knowledge GDR	2.97 (0.85)

### Study design

The survey consisted of three parts. In the first part, socio-demographic variables were collected. In the second part, the participants were randomly presented with one of four case vignettes. A vignette approach was chosen in which two persons with a GDR biography were created and varied with regard to traumatic experiences and injustice in the GDR. Following the presentation of the case vignette, various stigma-related questionnaires were specifically administered in reference to the case vignettes. In addition, stereotypes concerning about the described individuals in the two vignettes were also collected, which precede Link and Phelan’s (9) model. Finally, the participants were asked about their subjective knowledge of the GDR as it might be a possible confounding variable.

### Questionnaire

#### Case vignettes: life in the totalitarian state GDR with and without experiences of SED-injustice

Before randomization, all participants received a general case description. This depicted a person seeking counselling or treatment and described various psychosomatic complaints, such as mood swings, anxiety, headaches, and gastrointestinal complaints (see **Supplementary Appendix A**). After the general case description, participants received a randomized case vignette labelled according to sex and case version: case vignette A male (332 participants: male=161; female=171), case vignette B male (334 participants: male=162; female=172), case vignette A female (343 participants: male=166; female=177), case vignette B female (348 participants: male=163; female=185). Case vignette A described a person with an inconspicuous socialization in the GDR and a difficult life experience: traffic accident in which a friend died. Case vignette B, on the other hand, described a person, with a history of socialization in the GDR and an experience of political injustice in the GDR: denial of a place at university due to the parent’s activity in the political opposition, escape and political imprisonment and the resulting social, economic, and societal disadvantages.

#### Socio-demographic variables

In the present study, information on age and gender identity as well as the predominant location of socialization of the survey participants was requested. In addition, information was collected on the current profession (doctor, physiotherapist, speech therapist, occupational therapist, psychologist, psychotherapist, health and nursing professional) and the professional environment (inpatient vs. outpatient).

#### Subjective knowledge about the GDR and repression

Subjective knowledge about the GDR and repression, such as measures in connection with political persecution, was recorded using nine questions (e.g. knowledge about the GDR in general, the structure of the GDR home system or the various measures in connection with political persecution by the Ministry of State Security). The questions are based on the survey by Hoffmann and colleagues (45) and were formulated specifically for the context of GDR home education. These questions were expanded to include other experiences of injustice in the GDR (see **Supplementary Appendix B**). The assessment was based on a five-point Likert scale from *not present* (1) to *very good* (5). A mean score was calculated from this, with a high overall value indicating good subjective knowledge. The internal reliability was confirmed with a *Cronbach’s α* of.91. In addition, the participants were asked whether they had had contact with people who had experienced political injustice in the GDR in the course of their professional activities and whether they themselves had experienced political injustice at the hands of the SED.

### Attitudes toward people with mental disorders

#### Emotional reactions

The Emotional Reactions to Mental Illness Scale [ERMIS (60)] is a questionnaire that consists of ten items and can be summarized into three superordinate factors: *anger* (e.g. ‘I feel annoyed by this person’)*, fear* (e.g. ‘The person scares me’)*, pro-social reactions* (e.g. ‘I feel pity’). The items record the reactions that a person can show towards people with mental illness and are specifically related to the person described in the presented case vignette. The assessment was based on a five-point Likert scale from *strongly disagree* (1) to *strongly agree* (5). Higher values on the respective scale reflect a stronger emotional reaction towards the person described. The internal consistency is *Cronbach’s α* = .84 (fear), *Cronbach’s α* = .83 (anger) and *Cronbach’s α* = .65 (pro-social reaction). The ERMIS has already been used in studies involving employees in the healthcare profession (61). The mean score was calculated for each subscale and was used for the analyses.

#### Social distance

The Social Distance Scale (SDS) by Link and colleagues (62) was used to measure the desire for social distance from a person. The SDS consists of seven items that measure the willingness to engage in various everyday social situations (like renting a room, working together, or having as a neighbor) with the person described in the case vignette. The answers were assessed using a five-point Likert scale with the anchors *definitely* (1) and *definitely not* (5). Higher values on this scale indicate the desire for greater social distance from the person described. The internal consistency was *Cronbach’s α* = .84. The SDS has been used in several studies involving healthcare professionals (63–65). For the analyses, the mean score was calculated from all items.

#### Stereotypes towards a described person with mental health problems

In order to capture possible assumptions and prejudices about people with mental health problems, participants were asked to rate 14 statements (see **Supplementary Appendix B**) in relation to the person described in the case vignette. When creating the scale, we orientated ourselves on existing scales (stereotype of schizophrenia from: (66) and, Value-based Stigma Inventory from (67) and supplemented them with additional statements. These additions were reviewed and validated by the authors, who are also experts in this field. There were eight positive statements (e.g. “The person described is able to make good friends”) and six negative statements (e.g. “The person described is a danger to themselves and/or other people”). Participants were able to give their assessment using a five-point Likert scale from *strongly disagree* (1) to *strongly agree* (5). The internal consistency of the positive characteristics was *Cronbach’s α* =.81 and of the negative characteristics was *Cronbach’s α* = .76. A factor analysis carried out confirmed a two-factor structure (see **Table 2**). The mean value for each scale was calculated on the basis of the associated items, with higher values indicating a stronger expression of the respective scale.

**Table 2:** Factor structure of positive and negative stereotype.

Items	Factor loading	
	1	2
Factor 1: Positive Stereotype		
1. The person described will be able to recover.	**.61**	.11
2. The person described is as suitable for a responsible job as any other person.	**.69**	.04
3. The person described will continue to be able to make important decisions alone.	**.69**	-.06
4. The person described will continue to be able to lead a regular life.	**.74**	.00
5. The person described will be able to make good friends.	**.68**	.02
6. After treatment, the person described will be able to lead a normal life again.	**.61**	-.01
7. The person described fulfils their parental duties as well as other people.	**.73**	-.02
8. Basically, we all sometimes feel the same way as the person described.	**.46**	.16
Factor 2: Negative Stereotype		
9. The person described is to blame for their condition.	.02	**.76**
10. Treatment will do little to change the described person’s condition.	-.01	**.67**
11. The person described has an increased risk of committing suicide.	-.06	**.42**
12. The person described has an increased risk of drinking too much alcohol and/or taking drugs.	-.09	**.48**
13. The person described has a higher risk of becoming a criminal.	-.03	**.84**
14. The person described is a danger to themselves and/or other people.	-.04	**.79**

*N=1357*. The extraction method was principal axis factoring with a varimax rotation. Variance explained by both factors: 44.56% (Factor 1: 24.73%; Factor 2: 19.83%).

Bold values are factor loadings on the relevant factor.

### Statistical analyses

The statistical analyses were carried out using *SPSS* (version 29). In order to examine the differences between the stigma-relevant measures in relation to the two case vignettes, the differences in the mean values were calculated. Multiple regressions were performed using the bootstrap method with 1.000 iterations. The dependent variables included the mean score of the three ERMIS scales (anger, anxiety and pro-social reactions), SDS and the positive and negative stereotypes. The following variables were included in the multiple regression: socio-demographic characteristics (age, sex, West vs. East socialization), job-related aspects (occupation, professional environment), the version and sex of the case vignette as well as GDR-specific factors (contact with people who experienced SED injustice, own experiences of injustice and subjective knowledge about the GDR).

## Results

### Differences within the case vignette

With regard to the case vignettes (GDR socialization without injustice experience vs. experience of injustice in the GDR), the test for mean differences revealed significant differences in the scales desire for social distance, positive and negative assumption, and subjective knowledge about the GDR. Compared to case vignette A (no experience of injustice in the GDR), the participants showed higher desire for social distance (t(1355) = -4.92, *p* <.001, *d* = -0.27, 95% CI [-0.37,-0.16]), fewer positive (t(1355) = 7.67, *p* <.001, *d* = 0.42, 95% CI [0.31,0.52]), more negative assumptions (t(1355) = -6.27, *p* <.001, *d* = -0.34, 95% CI [-0.45,-0.23]) and less subjective knowledge about the GDR (t(1355) = -2.05, *p* <.001, *d* = -0.11, 95% CI [-0.22,-0.01]) for case vignette B (experience of injustice in the GDR). Further analyses also showed no significant differences between East and West socialization with regard to the stigma-relevant measures (ERMIS, SDS, positive and negative stereotypes), even taking into account the vignette version (GDR socialization without injustice experience vs. experience of injustice in the GDR).

### Multiple regressions for the prediction of stigmatizing attitudes

#### Emotional reaction fear

The multiple regression model shown in **Table 3** explains a total of 26.6% of the variance for the emotional reaction fear. Younger (*ß* = -.141; p < .001) and male participants showed a higher emotional response of fear (*ß* = -.218; p < .001), as did participants from the professional group of psychologists and psychotherapists (*ß* = .101; p < .001), professional group of occupational-, speech- and physiotherapist (*ß* = .095; p < .001), and working in an inpatient setting (*ß* = -.161; p < .001). Furthermore, a higher level of subjective knowledge about the GDR (*ß* = .228; p < .001) and who have an East German socialization (*ß* = .076; p < .01) had a significant influence on the regression model.

**Table 3:** Multiple regression (ERMIS Fear, Anger, Pro-Social).

Variable		ERMIS Fear		ERMIS Anger		ERMIS Pro-Social	
		*β*	*t*	*β*	*t*	*β*	*t*
Age		-.141***	-5.925	-.095***	-4.117	-.018	-0.663
sex (0=male, 1=female)		-.218***	-8.464	-.246***	-9.863	.062*	2.116
Socialisation (0=West Germany; 1= East Germany)		.076**	3.155	.052*	2.252	.021	0.758
Professional group Healthcare and nursing assistant (Reference)							
Medical doctor		-.011	-0.450	-.018	-0.781	-.022	-0.789
Psychologist/ Psychotherapist		.101***	-3.800	-.102***	3.974	.017	0.573
Other (Occupational therapist, Speech therapist, Physiotherapist, etc.)		.095***	3.668	.093***	3.715	-.005	-0.179
Work setting (0=outpatient, 1= inpatient)		-.161***	-6.249	-.161***	-6.415	-.060*	-2.036
Vignette version (0=Version A, 1=Version B)		.017	0.721	.026	1.140	.011	0.395
Vignette sex (0=male, 1=female)		-.031	-1.338	-.034	-1.496	-.049	-1.846
Subjective knowledge about the GDR		.228***	8.516	.267***	10.291	.181***	5.92
Contact with people with experience of SED injustice (0=yes 1=no)		.017	0.695	.004	0.156	-.024	-0.854
Own experience of SED injustice in the GDR (0=yes 1=no)		-.033	-1.371	-.003	-0.130	.004	0.127
Durbin Watson		1.774		1.757		1.937	
R²		0.266***		0.310***		0.037***	
Variable		Desire for Social Distance		Positive Stereotype		Negative Stereotype	
		*β*	*t*	*β*	*t*	*β*	*t*
Age		-.033	-1.222	.079**	2.980	-.152***	-6.641
sex (0=male, 1=female)		-.047	-1.615	.030	1.039	-.189***	-7.629
Socialisation (0=West Germany; 1= East Germany)		.051	1.900	-.032	-1.202	0.15	0.669
Professional Group Healthcare and nursing assistant (Reference)							
Medical doctor		.075**	2.775	-.018	-0.682	.019	0.808
Psychologist/ Psychotherapist		-.032	-1.059	.041	1.392	.094***	3.686
Other (Occupational therapist, Speech therapist, Physiotherapist, etc.)		-.039	-1.344	.012	0.424	.136***	5.488
Work setting (0=outpatient, 1= inpatient)		.038	1.326	-.050	-1.752	-.111***	-4.484
Vignette version (0=Version A, 1=Version B)		.145***	5.546	-.216***	-8.359	.151***	6.747
Vignette sex (0=male, 1=female)		-.040	-1.511	.016	0.610	-.061**	-2.733
Subjective knowledge about the GDR		-.212***	-7.073	.213***	7.174	.304***	11.823
Contact with people with experience of SED injustice (0=yes 1=no)		.020	0.706	.043	1.578	.011	0.443
Own experience of SED injustice in the GDR (0=yes 1=no)		-0.20	-0.746	-.027	-1.025	-.015	-0.654
Durbin Watson		1.893		1.865		1.712	
R²		0.075***		0.098***		0.323***	

* <0.05; ** <0.01; *** <0.001.

#### Emotional reaction anger

The multiple regression model (see **Table 3**) was able to account for a total of 31.0% of the variance regarding the emotional reaction anger. Male participants (*ß* = -.246; p < .001), the professional group of occupational-, speech- and physiotherapist (*ß* = .093; p < .001) and working in an inpatient setting (*ß* = -.161; p < .001) showed a stronger emotional reaction anger. The professional group of psychologists and psychotherapists showed fewer emotional anger reactions (*ß* =-.102; p<.001). In addition, male participants (*ß* = -.246; p = <.001) and a higher subjective knowledge about the GDR (*ß* = .267; p < .001) also predicted significantly stronger emotional reactions.

#### Emotional reaction pro-social reaction

The multiple regression model regarding the pro-social reactions of the ERMIS (see **Table 2**) explains 3.7% of the variance. Female participants (*ß* =.062; p<.05), participants who work in an outpatient setting (*ß* = -.060; p < .05) and those with a higher subjective knowledge about the GDR (*ß* = .181; p < .001) showed significantly greater pro-social reaction.

#### Desire for social distance

Overall, the presented regression model was able to explain 7.5% of the variance of the SDS. A strong predictor of the desire for social distancing was the version of the case vignette (case vignette B: *ß* = .145; p < .001). Medical doctors (*ß* = .075; p < .01) were the professional group that had an influence for desire for social distancing. The desire for social distancing was less pronounced if one had a high level of subjective knowledge about this topic (*ß* = -.212; p < .001).

#### Positive and negative stereotypes

Overall, the presented regression model was able to explain 9.8% of the variance of the positive and 32.3% of the variance of the negative stereotype. Older participants (*ß* = .079; p < .01), participants with higher subjective knowledge (*ß* = .213; p < .001) and those who were presented the case vignette with GDR socialization without experience of injustice (*ß* = -.216; p < .001) showed more positive stereotype attributions. In contrast, younger people (*ß* = -.152; p < .001), with high subjective knowledge (*ß* = .304; p < .001) men (*ß* = -.189; p < .001), working in an outpatient setting (*ß* = -.111; p < .05), male case vignette (*ß* = -.061; p < .01), and those who were presented the case vignette with experience of injustice (*ß* = .151; p < .001) showed more negative stereotypes attributions.

## Discussion

The aim of the current study was to investigate whether stigmatizing attitudes by professionals working in the healthcare system towards people with mental illness and a biography from the GDR-socialisation still exist three decades after the fall of Berlin Wall and the German reunification. The results indicate that it seems to make a difference whether one has experienced for the most part uncomplicated socialisation socialization or repression by the SED leadership in the past. Furthermore, sex and factors, such as the occupation and the treatment setting showed an influence on stigmatizing attitudes. The best predictive models were the emotional response of fear and anger and negative stereotypes.

### Manipulation of the case vignette

The multivariate analyses showed that the manipulation of the case vignette impacted the results. It was found that stigmatizing attitudes towards the case vignette with experiences of injustice in the GDR were more pronounced, whereby, the differences were small to moderate.

One possible explanation for the small/moderate differences could be the attribution of causes according to Weiner’s attribution-theory (53). In both case vignettes, an initial clinically relevant symptomatology was described. In the manipulation in the form of the biographical anamnesis, the overlaps between the two vignettes may have been too large. The described persons were both socialized in a similar way and in both cases experienced drastic events that influenced the further course of their lives differently. The person in case vignette A experienced an uncontrollable event for which they had no responsibility (uncontrollable, determined by others), and which largely had no far-reaching influence on their life. In the second vignette (B), the event was also primarily external (parents were in opposition), uncontrollable and stable in time. It was only later in life that the attribution shifted from external to internal (escape + political imprisonment) with the corresponding consequences for the rest of their life. It is possible that the causal attribution was similar in the perception in two of the three dimensions (external, uncontrollable).

Nevertheless, the case studies described two people with clearly recognizable mental distress and a corresponding need for treatment. In the present study, the average values of the stigma-relevant measures are even higher than in comparable vignette-based studies for stigmatising attitudes against people with mental disorders among professionals and in the general population (ERMIS: (60, 61, 68); SDS: (62, 63, 69, 70)). We can only hypothesize why the two case vignettes in our study were more stigmatized than in comparable studies. One possible explanation could be that the stronger stigmatization was due to GDR socialization. A recent study by the German Center for Integration and Migration Research (Berlin: 66) shows that people from Eastern-Germany (new federal states) are increasingly being treated in a similarly negative way by people from Western-Germany (old federal states) as people with a migration background. This can be seen, for example, in their portrayal as victims or the view that they have not arrived in current German society (71). Future research could investigate this by using a comparably neutral vignette.

### More knowledge means more stigmatizing attitudes?

Our survey showed that more subjective background knowledge about the GDR led to stronger emotional reactions such as fear and anger as well as negative stereotypes towards the people described. This contradicts the assumption from anti-stigma campaigns that imparting knowledge, for example through educational content, reduces stigmatizing attitudes (72, 73). On the other hand, however, more subjective knowledge about the GDR also led to stronger pro-social behaviour (emotional response), a lower desire for social distance and more positive stereotypes.

One possible explanation could be the dual-process model in connection with stigmatization processes (74–77). This model states that there are two types of reactions to stigmatization based on different knowledge systems. System One is a reflexive, initial response based on associative processes and instinctive, rapid emotional reactions. This occurs particularly when there are limited resources, time and motivation. On the other hand, system Two is a rule-based process that is slower and more controlled. It includes considerations about attributing causes and the resulting emotional reactions.

A comparison of the stigma-related questionnaires shows that the ERMIS in particular requires little cognitive capacity to answer the questions. This is due to the fact that the questions are designed more for initial reactions (e.g. “I feel uncomfortable” or “I react angrily”). Although existing knowledge about the GDR plays a role, it is mostly based on global knowledge about the GDR, which allows for less differentiated observations of the various case vignettes. In contrast, the questions in the SDS questionnaire and the stereotypes questionnaire are much more far-reaching and differentiated. Examples include questions such as: “To what extent would you accept the person described as a work colleague?” or “The person described poses a danger to themselves and/or other people.” These questions draw much more on existing knowledge in order to make a causal attribution.

With the appropriate knowledge, one could initially assume that someone is to blame for their situation, which, according to Weiner’s attribution theory, leads to an intuitive, angry reaction with correspondingly reduced support (see (54)). However, a differentiated view with more resources could reveal that although the person may possibly have some responsibility for their condition, their reaction to the further consequences may be of a prosocial nature (e.g. I feel compassion for the person to whom this has happened).

Initial evidence for this assumption comes from a study by Vitaglione and Barnett (78). This study showed that people can feel both sadness and anger in relation to an event in which someone was the victim of a drunk driver. At the same time, it was also shown that both emotions had a positive influence on possible helping behaviour. Interestingly, it was found that anger not only directly influenced helping behaviour, but also had a positive indirect effect on it via sadness. Another study by Fischer and Roseman (79) showed that an experience in which anger was felt both immediately and for some time afterwards can lead to reconciliation in the long term. This illustrates that different emotions can occur in relation to events and that these can be linked to helping behaviour.

### Influence of age and sex on stigmatizing attitudes

Our findings are in line with those of other studies that have shown that stigmatizing attitudes increase with age (80–82).

In our present study, male participants had stronger stigmatizing attitudes compared to female participants, especially with regard to the emotional reactions (fear and anger, ERMIS) and positive/negative stereotypes. The difference between the sex in relation to stigmatizing attitudes towards people with mental illness among students and healthcare professionals is shown in various studies. Male respondents stigmatize both mental illness in general and specific disorders (e.g. PTSD, depression, borderline personality disorder) more than women (83–86). Nevertheless, some studies also show that women in the healthcare sector show stronger stigmatizing attitudes towards people with mental illness in general (87) and especially towards people with addiction disorders (84).

Some mental disorders (Antisocial Personality Disorder, Schizophrenia, Substance-Use-Disorder vs. Anxiety-Disorders) are subject to stronger stereotyping processes compared to others and that this plays a role in the classification of the results of the present study (59, 88). Boysen and colleagues (89) conducted several studies to investigate whether certain mental disorders can be classified into masculine and feminine categories and how people perceive these disorders in the context of stigmatizing attitudes. The results suggest that there are some disorders which are stereotypically associated with masculinity (e.g. paedophilia, addictions, schizophrenia, antisocial personality disorder) and femininity (e.g. depression, eating disorders, anxiety disorder, borderline personality disorder). However, these stereotypical categorizations of mental disorders differ in terms of the degree of stigmatization, with male-categorized disorders being more stigmatized than female-categorized disorders (89–91). In the present study, vignettes with two biographical scenarios were presented, each of these scenarios dealing with possible traumatic events (car accident resulting in death vs. political imprisonment). These scenarios correspond to the classification (post-traumatic stress disorder) of mental disorders related to masculinity (89). This is also in line with the research findings of Kaitz and colleagues (85), who showed a sex difference in people with post-traumatic stress disorder in relation to stigmatizing attitudes of people from the healthcare system, where male healthcare providers showed higher stigmatizing attitudes than female. In the current study, two vignettes described a traumatic experience that participants associated with the symptoms of post-traumatic stress disorder.

### Influence of job-related factors

Our study shows that staff in outpatient facilities have less stigmatizing attitudes than their counterparts in inpatient facilities, which is consistent with other research findings (92, 93). A potential explanation could be that more severe, complex cases and acute crises tend to be treated preferably in an inpatient setting and thus the severity of symptoms associated with acute crises leads to increased stigmatizing attitudes by professionals (92, 93). On the other hand, various studies suggest that direct contact with people suffering from mental illness helps to reduce the stigmatizing attitudes of professionals (for an overview (94)). Particularly in the inpatient sector, the organisation of the general conditions leads to more intensive and closer contact than in the outpatient sector. At the same time, the usually less severe nature of symptoms offers the opportunity to become better familiar with the people affected and their illness, which in turn can have a reducing effect on stigmatising attitudes.

The finding that psychologists/psychotherapists and doctors tend to have more stigmatizing attitudes than other groups of health care professionals, although the correlations in our results are low, fits into the picture of the heterogeneous findings between the different professional groups. Several review articles show that there are stigmatizing attitudes among therapists (95, 96). Various studies have shown that healthcare professionals have less stigmatizing attitudes than the general population (97, 98), although some study showed psychiatrists have more stigmatizing attitudes than psychologist and the general population (99, 100). A study by Masedo and colleagues (101) demonstrated that psychology and occupational therapy students exhibited lower levels of stigmatizing attitudes compared to their counterparts in medicine and nursing.

### Strengths and limitation

This is the first vignette-based study on the stigmatization of people with mental health issues from the former GDR who experienced SED injustice by medical professionals. An online survey with over 1000 healthcare participants makes it a major survey on stigmatizing attitudes toward mental illness, benefiting from high respondent availability. In the recent years, the participation of both participants and researchers in various online platforms for conducting surveys has clearly increased (102, 103). This development offers the advantage of wide availability of participants, which can help to reduce costs and time compared to postal surveys (104, 105). However, online surveys are also associated with some disadvantages. In addition to quality issues, panel survey providers are increasingly encountering differences in data quality (103, 106). Compared to other survey methods such as face-to-face interviews or paper surveys, the response rate for online surveys is lower (107, 108). In online surveys, participants frequently exhibit selectivity based on various factors such as internet access, familiarity with the surveys, self-motivation, and incentives for participation. This selectivity, as highlighted by Bethlehem (109), results in a sample that is not representative, posing challenges in generalizing the findings, as noted by Greenacre (110). Data quality was ensured by filtering out identical sentences in open questions chatbot responses. Nurses were overrepresented, while doctors and therapists were underrepresented, but statistical significance for the healthcare sector remains. Differences between professional groups were small, and the workplace (psychiatric vs. somatic) was not considered. Additionally, the sample size for individual medical disciplines was too small to analyse subgroup differences.

It also remains unclear whether the respondents have direct contact with people with mental illness, as this was not included in the survey, although studies suggest that such contact reduces stigmatising attitudes (84, 111).

### Implications for practice

Over the past two decades, interest in the stigmatisation of mental illness has grown continuously, which can be clearly seen in the increasing number of publications (25, 112, 113). In recent years, stigmatising attitudes among professionals have decreased (meta-analysis by (114)). Nevertheless, our study highlights the need for anti-stigma interventions in healthcare in general and in a specific context with people who experienced SED-injustice such as in the present study. Therefore, we suggest introducing various stigma reduction interventions (education, face-to-face contact or ideally a combination of both) to raise awareness at different levels (stereotypes, prejudice, discrimination) at an early stage, which have been shown to be effective in various reviews and meta-analyses (72, 94, 115). Based on the model by Link and Phelan (9) and its extension (10), studies show that emotional reactions lead to the desire for social distancing (60, 97, 116).

In Pryor and colleagues’ model (75), emotional reactions are considered as System One (reflexive processes, instinctive emotional reactions) and the desire for social distance as System Two (rule-based processes: controlled processes, attributional reasoning, derived emotional reactions). This highlights that interventions should target System Two in particular and focus on rule-based processes (77). Classic stigma prevention strategies such as protest, education and interpersonal contact (117) mainly affect System Two. Interpersonal contact can also influence System One (stereotypes) by establishing emotional connections with those affected (77). Studies show that emotional reactions (System One) such as anger and compassion mediate the relationship between stereotypes (System One) and behaviour (System Two). Uncontrollable events lead to more compassion and helpfulness, and controllable events lead to more anger and more aggressive behaviour (54). Further studies on anxiety disorders, Alzheimer’s disease and schizophrenia demonstrate that compassion, fear and anger influence the relationship between stereotypes and discriminatory behaviour (118, 119). On the other hand, a longitudinal study by Koike and colleagues (120) shows the significant influence of stereotypes on discriminatory behaviour. While, in line on expectations, the initial level of desire for social distance predicts the level of social distance after twelve months, negative stereotypes increase this desire for social distance both at the beginning and over time. This makes it clear that both systems need to be influenced by different approaches to anti-stigma interventions.

## Conclusion

More than 30 years after reunification, people who experienced injustice at the hands of the SED continue to experience stigmatisation, which can be a potential obstacle to seeking medical or psychotherapeutic help. Many of those affected report a lack of understanding on the part of those treating them and difficulties in finding suitable therapy programmes that take their specific experiences into account. Furthermore, mistrust of institutions, fear of re-traumatisation and a lack of social reappraisal of this past can make it even more difficult to seek support.

In view of current religious and political conflicts, for example in Ukraine and the Middle East, as well as the situation of political prisoners in countries such as Iran and China, there will continue to be people who experience injustice and are dependent on support from the healthcare system. Traumatic experiences caused by state repression, war or persecution require sensitive and specialised treatment in order to do justice to the particular burdens of those affected. The results presented here could therefore also be transferable to these groups of people and provide important insights for the future treatment of the health consequences of political persecution and systematic injustice.

## Data Availability

The datasets presented in this article are not readily available because the data sets compiled and/or analysed as part of the study are not publicly accessible due to their sensitivity (e.g. due to reports of personal experiences of injustice). Requests to access the datasets should be directed to Tobias.Schott@medizin.uni-leipzig.de.
