# Correction: Stigmatisation of survivors of political persecution in the GDR: attitudes of healthcare professionals

**DOI:** 10.3389/fpsyt.2025.1657776

**Published:** 2025-09-03

**Authors:** Tobias Schott, Marie Blume, Anne Weiß, Christian Sander, Georg Schomerus

**Affiliations:** Department of Psychiatry and Psychotherapy, University of Leipzig Medical Centre, Leipzig, Germany

**Keywords:** GDR, SED, reunification, mental disorders, attitude, stigma, marginalization, health-care system

There was a mistake in **Table 1** as published. An incorrect distribution of values was entered in the section *Work Setting*. The corrected **Table 1** appears below.

**Table 1:** Sociodemographic variables.

Variable	Mean *(SD)* or % (*n*) (*N*=1357)
Age (years)	43.14 (10.86)
Sex	
Male	48.0% (652)
Female	52.0% (705)
Primarly socialization	
West-Germany	60.9% (826)
East-Germany	34.7% (471)
Outside of Germany	4.4% (60)
Professional group	
Medical doctor	8.7% (118)
Healthcare and nursing assistant	64.4% (874)
Psychologist/Psychotherapist	14.7% (200)
Other (Occupational therapist, Speech therapist, Physiotherapist, etc.)	12.2% (165)
Work setting	
Outpatient	45.2% (614)
Inpatient	54.8% (743)
Contact with people with experience of SED injustice	
Yes	28.62% (388)
No	71.4% (969)
Own experience of SED injustice in the GDR	
Yes	3.1% (42)
No	96.9% (1315)
ERMIS	
Fear	1.93 (0.99)
Anger	1.81 (1.01)
Pro-social behavior	3.63 (0.78)
Social Distance Scale	2.63 (0.77)
Positive Stereotype	3.53 (0.68)
Negative Stereotype	2.83 (0.69)
Subjective Knowledge GDR	2.97 (0.85)

There was a mistake in **Table 3** as published. The values in the published table have changed due to other calculations with a larger data set. The corrected **Table 3** appears below.

**Table 3:** Multiple regression (ERMIS Fear, Anger, Pro-Social).

Variable		ERMIS Fear		ERMIS Anger		ERMIS Pro-Social	
		*β*	*t*	*β*	*t*	*β*	*t*
Age		-.141***	-5.925	-.095***	-4.117	-.018	-0.663
sex (0=male, 1=female)		-.218***	-8.464	-.246***	-9.863	.062*	2.116
Socialisation (0=West Germany; 1= East Germany)		.076**	3.155	.052*	2.252	.021	0.758
Professional group Healthcare and nursing assistant (Reference)							
Medical doctor		-.011	-0.450	-.018	-0.781	-.022	-0.789
Psychologist/ Psychotherapist		.101***	-3.800	-.102***	3.974	.017	0.573
Other (Occupational therapist, Speech therapist, Physiotherapist, etc.)		.095***	3.668	.093***	3.715	-.005	-0.179
Work setting (0=outpatient, 1= inpatient)		-.161***	-6.249	-.161***	-6.415	-.060*	-2.036
Vignette version (0=Version A, 1=Version B)		.017	0.721	.026	1.140	.011	0.395
Vignette sex (0=male, 1=female)		-.031	-1.338	-.034	-1.496	-.049	-1.846
Subjective knowledge about the GDR		.228***	8.516	.267***	10.291	.181***	5.92
Contact with people with experience of SED injustice (0=yes 1=no)		.017	0.695	.004	0.156	-.024	-0.854
Own experience of SED injustice in the GDR (0=yes 1=no)		-.033	-1.371	-.003	-0.130	.004	0.127
Durbin Watson		1.774		1.757		1.937	
R²		0.266***		0.310***		0.037***	

Variable		Desire for Social Distance		Positive Stereotype		Negative Stereotype	
		*β*	*t*	*β*	*t*	*β*	*t*
Age		-.033	-1.222	.079**	2.980	-.152***	-6.641
sex (0=male, 1=female)		-.047	-1.615	.030	1.039	-.189***	-7.629
Socialisation (0=West Germany; 1= East Germany)		.051	1.900	-.032	-1.202	0.15	0.669
Professional Group Healthcare and nursing assistant (Reference)							
Medical doctor		.075**	2.775	-.018	-0.682	.019	0.808
Psychologist/ Psychotherapist		-.032	-1.059	.041	1.392	.094***	3.686
Other (Occupational therapist, Speech therapist, Physiotherapist, etc.)		-.039	-1.344	.012	0.424	.136***	5.488
Work setting (0=outpatient, 1= inpatient)		.038	1.326	-.050	-1.752	-.111***	-4.484
Vignette version (0=Version A, 1=Version B)		.145***	5.546	-.216***	-8.359	.151***	6.747
Vignette sex (0=male, 1=female)		-.040	-1.511	.016	0.610	-.061**	-2.733
Subjective knowledge about the GDR		-.212***	-7.073	.213***	7.174	.304***	11.823
Contact with people with experience of SED injustice (0=yes 1=no)		.020	0.706	.043	1.578	.011	0.443
Own experience of SED injustice in the GDR (0=yes 1=no)		-0.20	-0.746	-.027	-1.025	-.015	-0.654
Durbin Watson		1.893		1.865		1.712	
R²		0.075***		0.098***		0.323***	

* <0.05; ** <0.01; *** <0.001.

In the **Abstract**, in the *Results* section, two values were incorrect. This section originally read, “The explanatory power of the regression models is predominantly in the medium range (from 9.7 till 35.3%).” This has been corrected to read:

“The explanatory power of the regression models is predominantly in the medium range (from 3.7 till 32.3%).”

In the **Results** section, the values have been adjusted according to the changes in **Table 3**.

The subsection *Emotional reaction fear* originally read, “The hierarchical regression model shown in **Table 3** explains a total of 28.0% of the variance for the emotional reaction fear. Younger (ß = -.100; p <.01) and male participants showed a higher emotional response of fear (ß = -.204; p <.001), as did participants from the professional group of psychologists and psychotherapists (ß = .130; p <.001), professional group of occupational-, speech- and physiotherapist (ß = .168; p <.001), and working in an inpatient setting (ß = -.155; p <.001). Furthermore, a higher level of subjective knowledge about the GDR (ß = .182; p <.001) and an own experience of SED injustice (ß = -.065; p <.05) had a significant influence on the regression model.” This has been corrected to read:

“The multiple regression model shown in **Table 3** explains a total of 26.6% of the variance for the emotional reaction fear. Younger (ß = -.141; p < .001) and male participants showed a higher emotional response of fear (ß = -.218; p < .001), as did participants from the professional group of psychologists and psychotherapists (ß = .101; p < .001), professional group of occupational-, speech- and physiotherapist (ß = .095; p < .001), and working in an inpatient setting (ß = -.161; p < .001). Furthermore, a higher level of subjective knowledge about the GDR (ß = .228; p < .001) and who have an East German socialization (ß = .076; p < .01) had a significant influence on the regression model.”

The subsection *Emotional reaction anger* originally read, “The multiple hierarchical regression model (see **Table 3**) was able to account for a total of 35.0% of the variance regarding the emotional reaction anger. Male participants (ß = -.225; p < .001), the professional group of psychologists and psychotherapists (ß =.138; p<.001) as well as professional group of occupational-, speech- and physiotherapist (ß = .186; p < .001) working in an inpatient setting (ß = -.157; p < .001) and having personally experienced SED injustice (ß = -.071; p = .024) showed a stronger emotional reaction anger. In addition, the sex form of the case vignette (ß = .083; p = .006) and a higher subjective knowledge about the GDR (ß = .208; p < .001) also predicted significantly stronger emotional reactions.” This has been updated to read:

“The multiple regression model (see **Table 3**) was able to account for a total of 31.0% of the variance regarding the emotional reaction anger. Male participants (ß = -.246; p < .001), the professional group of occupational-, speech- and physiotherapist (ß = .093; p < .001) and working in an inpatient setting (ß = -.161; p < .001) showed a stronger emotional reaction anger. The professional group of psychologists and psychotherapists showed fewer emotional anger reactions (ß =-.102; p<.001). In addition, male participants (ß = -.246; p = <.001) and a higher subjective knowledge about the GDR (ß = .267; p < .001) also predicted significantly stronger emotional reactions."

The subsection *Emotional reaction pro-social reaction* originally read, “The multiple hierarchical regression model regarding the pro-social reactions of the ERMIS (see **Table 3**) explains 9.7% of the variance. The professional group of psychologists and psychotherapists (ß =.082; p<.05) as well as the professional group of occupational-, speech- and physiotherapist (ß = .099; p < .01) and those with a higher subjective knowledge about the GDR (ß = .225; p < .001) showed significantly greater pro-social reaction.” This has been updated to read:

“The multiple regression model regarding the pro-social reactions of the ERMIS (see **Table 2**) explains 3.7% of the variance. Female participants (ß =.062; p<.05), participants who work in an outpatient setting (ß = -.060; p < .05) and those with a higher subjective knowledge about the GDR (ß = .181; p < .001) showed significantly greater pro-social reaction.”

The subsection *Desire for social distance* originally read, “Overall, the presented hierarchical regression model was able to explain 10.3% of the variance of the SDS. The strongest predictor of the desire for social distancing was the version of the case vignette (ß = -.140; p < .001). Medical doctors (ß = .131; p < .001) and the group of occupational-, speech- and physiotherapist (ß = -.123; p < .01) were the professional group that had an influence for desire for social distancing. The desire for social distancing was less pronounced if one had already had contact in a professional context with people who had experienced injustice in the GDR (ß = .104; p < .01) and if one had a high level of subjective knowledge about this topic (ß = -.150; p < .001).” This has been updated to read:

“Overall, the presented regression model was able to explain 7.5% of the variance of the SDS. A strong predictor of the desire for social distancing was the version of the case vignette (case vignette B: ß = .145; p < .001). Medical doctors (ß = .075; p < .01) were the professional group that had an influence for desire for social distancing. The desire for social distancing was less pronounced if one had a high level of subjective knowledge about this topic (ß = -.212; p < .001).”

The subsection *Positive and negative stereotypes* originally read, “Overall, the presented regression model was able to explain 12.6% of the variance of the positive and 35% of the variance of the negative stereotype. Older participants (ß = .118; p < .001), participants with higher subjective knowledge (ß = .172; p < .001) and those who were presented the case vignette with GDR socialization without experience of injustice (ß = .225; p < .001) showed more positive stereotype attributions. In contrast, younger people (ß = .115; p < .001), with high subjective knowledge (ß = .115; p < .001) men (ß = .115; p < .001), working in an outpatient setting (ß = .087; p < .05), and those who were presented the case vignette with experience of injustice (ß = .115; p < .001) showed more negative stereotypes attributions.” This has been updated to read:

“Overall, the presented regression model was able to explain 9.8% of the variance of the positive and 32.3% of the variance of the negative stereotype. Older participants (ß = .079; p < .01), participants with higher subjective knowledge (ß = .213; p < .001) and those who were presented the case vignette with GDR socialization without experience of injustice (ß = -.216; p < .001) showed more positive stereotype attributions. In contrast, younger people (ß = -.152; p < .001), with high subjective knowledge (ß = .304; p < .001) men (ß = -.189; p < .001), working in an outpatient setting (ß = -.111; p < .05), male case vignette (ß = -.061; p < .01), and those who were presented the case vignette with experience of injustice (ß = .151; p < .001) showed more negative stereotypes attributions.”

The original version of this article has been updated.

