# Supplementary material for: Stigmatisation of survivors of political persecution in the GDR: attitudes of healthcare professionals
Source: Front Psychiatry. 2025 Jun 10;16:1556411. doi: 10.3389/fpsyt.2025.1556411 (PMC12185403; doi:10.3389/fpsyt.2025.1556411)
Supplement: Supplementary file 2 [file Table1.docx]

Appendix A

Vignetteforms translated from German

Imagine that the following patient comes for treatment and describes his or her complaints:

The patient presents because of various physical and psychological complaints. He/she has been suffering from recurring headaches, shoulder and back pain, nausea, malaise and gastrointestinal complaints for years. In recent months, he/she has repeatedly struggled with significant mood swings due to retirement. The patient feels little joy, has little appetite, has difficulty sleeping and always seems jumpy, tense and restless, with an evasive gaze. In various situations in which the patient finds him/herself (e.g. large gatherings of people), anxiety and panic attacks occur repeatedly.

Case A:

From the biographical history, you learn that the patient is 65 years old, was born in the GDR and grew up there. He/she had a normal childhood and youth and lived with his/her parents in a small village. There had been a good connection to the village community. In young adulthood, the patient completed a teaching degree and enjoyed working there for many years. During his studies, the patient was a passenger in a serious traffic accident in which one of his two friends was killed. After graduating, he/she met his/her current wife/husband at work. The family live together in a house and have two children. Overall, he/she was able to lead a good life in the GDR. The patient had little interest in political issues. He/she experienced the period of reunification without any major difficulties. Their children have since left home. The couple continue to live in the house they share, are financially well off and are well integrated in the neighbourhood.

Case B:

From the biographical history, you learn that the patient is 65 years old, was born in the GDR and grew up there. His/her childhood and youth were not easy. His/her parents were politically active in the opposition. As a result, the family had not been able to make many friends in the neighbourhood. As a young adult, the patient had wanted to start university. Despite good school results, he/she was refused. Instead, he/she started an apprenticeship in quality control, but dropped out. Due to the lack of future prospects, the patient had decided to flee the GDR, but had been apprehended in the process. As a result of the escape attempt, he/she had spent several months in political prison. During this time, he/she had experienced some difficult situations. After imprisonment, the patient had had difficulties gaining a foothold professionally and in the community. After reunification, the professional and social situation had changed only slightly. He/she had received social welfare for a long time. The financial situation is strained, the patient feels left behind and is socially isolated.

Appendix B

| Items Subjective Knowledge about the GDR | |
| --- | --- |
|  |  |
| 1. How would you rate your general level of knowledge about the GDR? | |
| 1. Various measures in connection with political persecution by the Ministry of State Security (e.g. psychiatrisation, home education, subversion, systematic discrediting, humiliation, stigmatisation, disinformation, interrogation methods and examination conditions, etc.). | |
| 1. Pedagogical and educational principles of the GDR era (theories and scientific findings that formed the basis for home education, among other things) | |
| 1. Organisation of the GDR home system with different types of care homes (normal care home, transit care home, special children's care home, youth work camp, etc.) | |
| 1. State doping in the GDR in the field of professional sport | |
| 1. Incorrect medical treatment (e.g. hepatitis C-contaminated anti-D immunoprophylaxis for women) | |
| 1. Child abduction/forced adoption | |
| 1. Forced treatment/psychiatrisation | |
| 1. How would you rate your general level of knowledge about the GDR? | |
|  | |
